# Demonstrating the effectiveness of intra-articular prolotherapy combined with peri-articular perineural injection in knee osteoarthritis: a randomized controlled trial

**DOI:** 10.1186/s13018-024-04762-4

**Published:** 2024-05-05

**Authors:** Yiling Fu, Yukun Du, Jianyi Li, Yongming Xi, Wenbin Ji, Tieshan Li

**Affiliations:** 1 Qilu Hospital of Shandong University (Qingdao), Qingdao, China; 2 The Affiliated Hospital of Qingdao University, Qingdao, China

**Keywords:** Knee osteoarthritis, Prolotherapy, Peri-articular perineural injection, VAS

## Abstract

**Background:**

This study aimed to compare the efficacy of intra-articular prolotherapy (IG) combined with peri-articular perineural injection (PG) in the management of knee osteoarthritis (KOA).

**Methods:**

A total of 60 patients with the diagnosis of KOA were included in this double-blinded randomized controlled clinical trials. The inclusion criteria were as follow: (1) 48–80 years old; (2) the diagnose of KOA; (3) the grade 2 and 3 of the Kellgern–Lawrence grading scale; (4) the pain, crepitation, and knee joint stiffness continuing for 3 months at least. The main exclusion criteria were as follow: (1) any infection involving the knee skin; (2) history of any Influencing factors of disease. All patients were divided into three groups and received either IG, PG and I + PG under the ultrasound guidance and the 2, 4 and 8 weeks follow-up data of patients were available. (IG n = 20 or PG n = 20, I + PG n = 20). Visual Analogue Scale (VAS), The Western Ontario McMaster University Osteoarthritis Index (WOMAC) and the pressure pain threshold (PPT) were used as outcome measures at baseline, 2, 4 and 8 weeks.

**Results:**

There were no statistically significant differences in terms of age, sex, BMI, duration of current condition and baseline assessments of pain intensity, WOMAC scores and PPT. After treatment, the improvement of VAS activity, WOMAC and PPT values was showed compared with pre-treatment in all groups (*p* < 0.05). At 4 and 8 weeks after treatment, the VAS and WOMAC scores of the I + PG were significantly lower than those of the PG or IG, and the difference was statistically significant (*p* < 0.05). The PPT values of PG and I + PG were significantly improved compared to IG at 2, 4, and 8 weeks after treatment.

**Conclusion:**

The ultrasound guided I + PG of 5% glucose seem to be more effective to alleviate pain and improve knee joint function than single therapy in short term. Clinical rehabilitators could clinically try this combination of I + PG to improve clinical symptoms in patients with KOA.

## Introduction

Knee osteoarthritis (KOA) is one of the common degenerative disease in middle aged and elderly people, the incidence of KOA increased significantly with age [1]. KOA not only affects the life quality of patients, but also improves major economic burdens on the society [2]. The emerging evidence suggests that KOA affects intra and extra articular structures, such as the articular cartilage, subchondral bone, articular capsule, synovium, meniscuses, tendons, and ligaments [3]. The main symptoms are joint pain, joint stiffness and decreased function [4]. Pain is the most common complaint that forces patients to seek treatment with the goals of relieving symptoms, improving functional status and the quality life of patients [5].

Based on the severity of the disease, a series of treatment options can be accepted range from conservative treatment to surgical techniques [6]. There are many non-operative treatments including oral analgesia, physical therapy, multiple injections and so on [5]. However, due to the complex and unclear pain mechanism of KOA, the efficacy of these treatments still cannot meet the clinical needs, and even serious adverse reactions occur [7]. Although surgery is more effective, studies have shown that up to 20% of patients still have knee pain after surgery, and 2% of patients will have serious postoperative complications [8]. Intra-articular injection is an effective method for the treatment of osteoarthritis. Through puncture, the effusion was removed and drugs were injected into the intra-articular cavity which can effectively relieve the pain, improve the joint function and the quality of patient’s life. At present, the injection drugs of KOA mainly include sodium hyaluronate, glucocorticoids, platelet-rich plasma, botulinum toxin and ozone. Some scholars have also conducted clinical comparative experiments of those drugs, aiming to study the clinical efficacy of different drugs in the treatment of KOA [9–11].

In recent years, the prolotherapy is one of the new methods to treat chronic musculoskeletal pain by injecting small doses of irritating solution into the joint cavity or ligament to repair damaged or degraded cartilage, ligaments and other joint supporting structures [12]. Due to the low price and high safety, the hypertonic dextrose has become the most widely used injection drug in prolotherapy. The studies showed that hypertonic glucose can cause inflammation in cells and trigger an inflammatory cascade [13]. In addition, it theoretically increases growth factors and cytokines, thereby improving soft tissue healing and joint function [12]. However, OA-related pain is a specific condition with complex pathophysiology. Both knee structural lesions and neurological changes play an important role in the pain mechanism of KOA, which often results in limited efficacy of prolotherapy and cannot achieve complete pain relief, so it is necessary to find a combination therapy to achieve better results [14].

Perineural injection is a therapy for chronic neuropathic pain, which inject 5% dextrose into the painful nerve to achieve pain relief [15]. Researchers have used this therapy to inject around periarticular sensory nerves, with the fascial penetration point of the subcutaneous plane, and may be more effective than intra-articular prolotherapy [16]. The mechanism is hypothesized to be the inhibition of the expression of transient receptor potential vanilloid 1 (TRPV1) receptors leading to the release of substance P and calcitonin gene related peptide (CGRP) to reduce neurogenic inflammation in the joints [17].

To our knowledge, intra-articular hypertonic dextrose prolotherapy is a safe and effective non-surgical option for KOA [18]. But, there was no study on comparisons between intra-articular versus extra-articular dextrose injections or combination therapy to see if combination therapy achieves better outcomes, so we designed this study to investigate the effectiveness of three treatment options on decreasing pain and improving functions of knee osteoarthritis patients.

## Methods

The study was reviewed and approved by the Ethics Committee of the Hospital on 18th June 2022. All procedures were in accordance with the ethical standards of the institutional and/or national research committee. All patients were given informed consent in writing prior to inclusion in the study.

### Patient selection

**Fig. 1:**
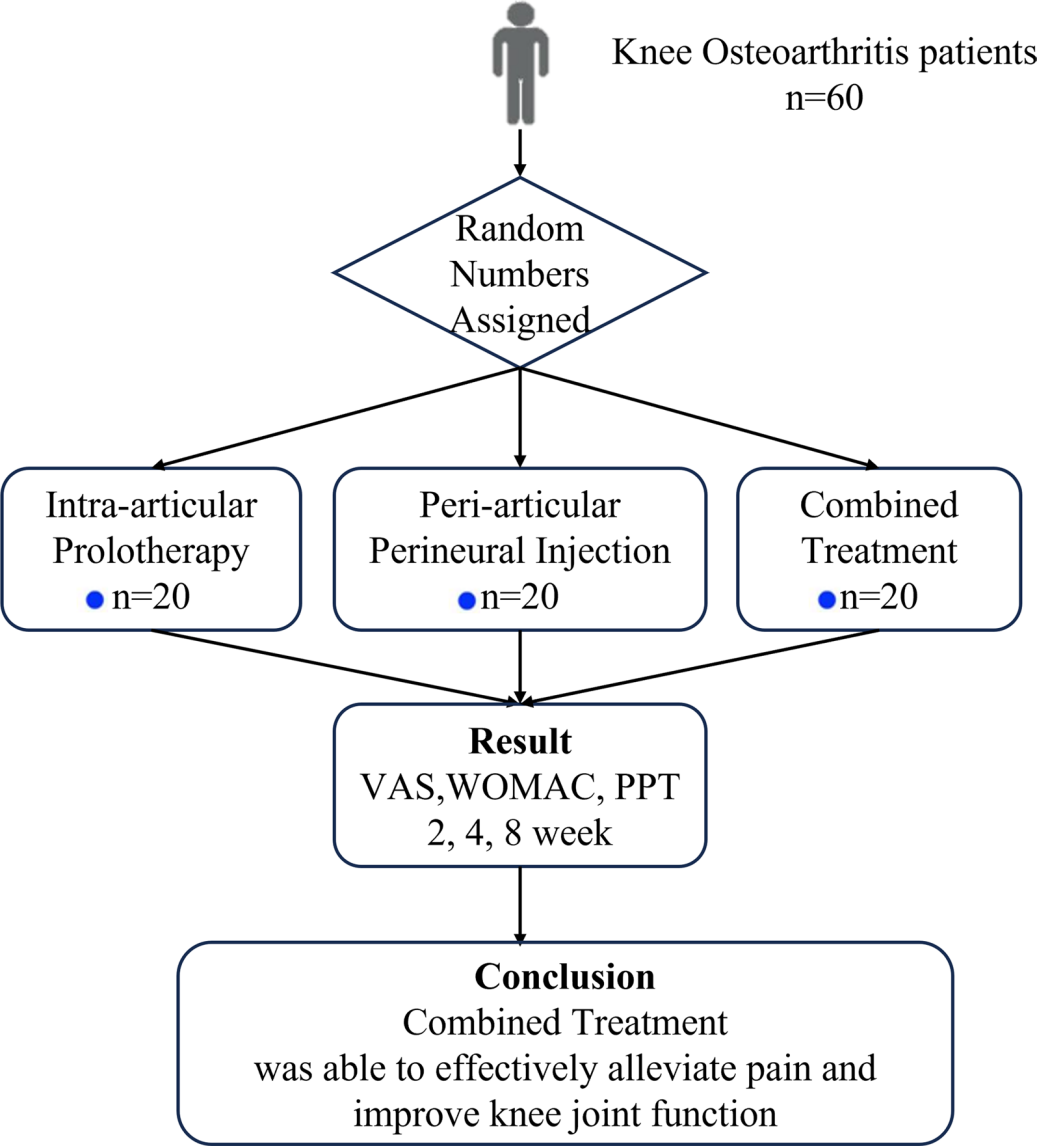
The flowchart of this study

This study was the randomized controlled clinical trials. Total of 60 KOA patients were enrolled in the study. The subjects were divided into three groups based on random numbers assigned by computer. The SPSS Visual Binning function was used to helps us quickly group. Participants were randomly assigned to intra-articular prolotherapy group (IG), Peri-articular perineural injection group (PG) or combined treatment group (I + PG). The physicians of conducting injection and evaluation were blind to the subject groups. (Fig. 1).

The inclusion criteria were as follow: (1) patient’s age was of 48–80 years old; (2) the diagnose of KOA; (3) the grade 2 and 3 of the Kellgern–Lawrence grading scale; (4) the pain, crepitation, and knee joint stiffness continuing for three months at least.

The exclusion criteria were as follow: (1) any infection involving the knee skin; (2) history of knee tumors or tuberculosis; (3) any intra- or peri-articular injection during the three last months; (4) prior total knee arthroplasty; (5) history of acute lumbosacral radiculopathy or peripheral neuropathy, bleeding disorders and pregnancy.

### The treatment procedure of three groups

All injections were performed by using ultrasound guided (4–18 Hz linearprobe, Mindray, Crius MEBP). Injections were repeated at 2 and 4 weeks after the first injection. All analgesics were discontinued 2 days before and 2 weeks after injection. All injections were conducted by the same physician.

#### Intra-articular prolotherapy injection

**Fig. 2:**
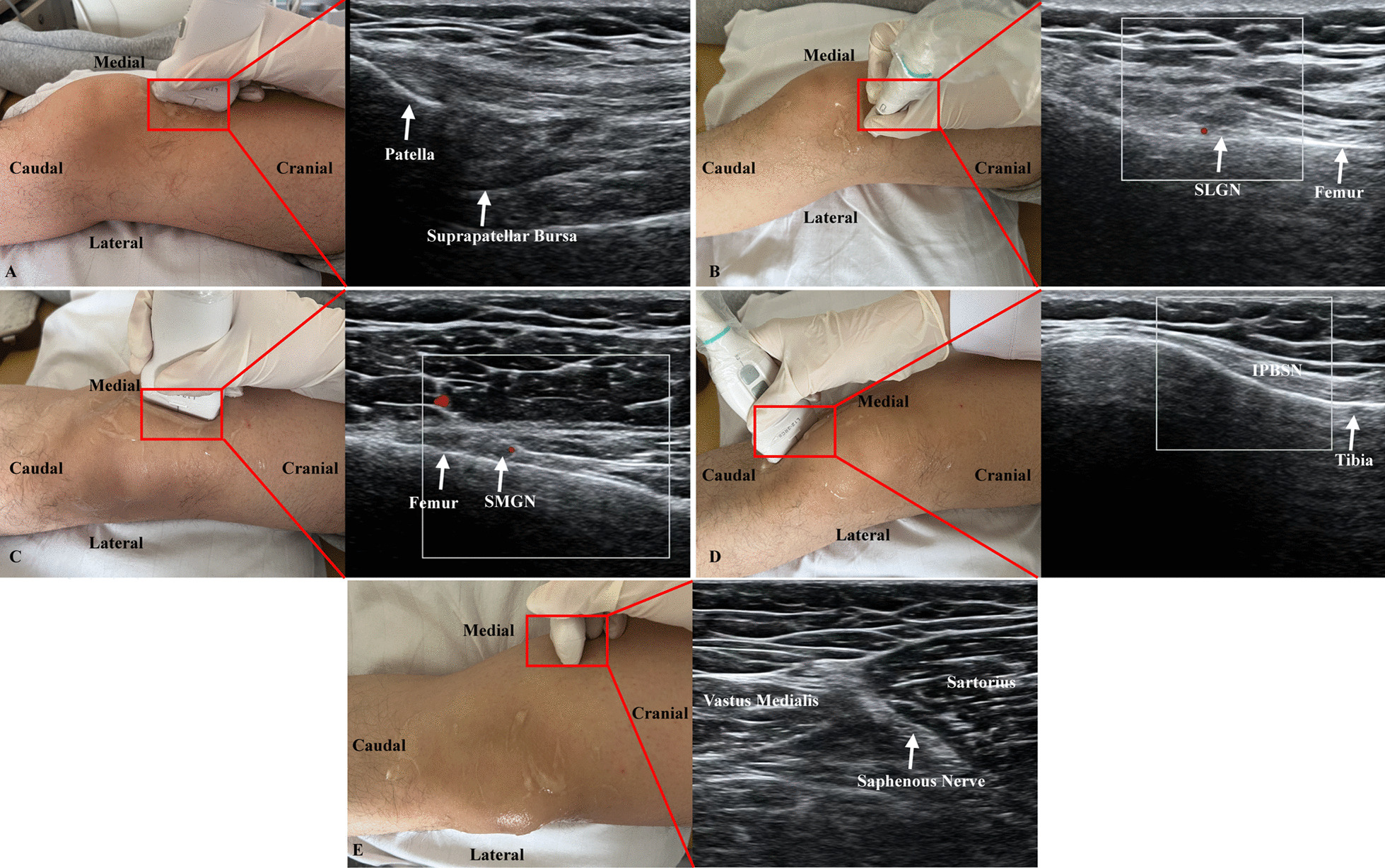
The location of** A** suprapatellar bursa and 4 deep nerve branches including **B** superolateral genicular nerve (SLGN), **C** superomedial genicular nerve (SMGN), infrapatellar branch of saphenous nerve (IPBSN), and **E** saphenous nerve

The patient was placed in a supine position with knee flexion of 20°–30°, and the injection site and surrounding skin were disinfected. A high-frequency ultrasound probe under musculoskeletal conditions was placed near the lower femur and above the patella to find the suprapatellar bursa (Fig. 2). The intra-articular prolotherapy injection group (IG) participant was injected (25-gauge needle) 8 mL of the 20% dextrose with assistance of ultrasound guidance.

#### Peri-articular perineural injection

After referring the former studies, the peri-articular perineural injection group (PG) participant was located 4 deep nerve branches including saphenous nerve, infrapatellar branch of saphenous nerve (IPBSN), superomedial genicular nerve (SMGN) and superolateral genicular nerve (SLGN) by using ultrasound guidance [19, 20]. Total of 2 mL of 5% glucose was injected (25-gauge needle) around each nerve under the real-time guidance of ultrasound images (Fig. 2).

Intra-articular prolotherapy injection combined with Peri-articular perineural injection.

The intra-articular prolotherapy injection combined with Peri-articular perineural injection (I + PG) participant was treated with the combination of IG and PG.

### Outcome measures

Baseline demographic criteria included age, BMI, sex and duration of current condition. Pain intensity was measured with Visual Analogue Scale (VAS) [21]. The Western Ontario and McMaster Universities arthritis index (WOMAC) was used to evaluate the patient’s knee function [22]. The pressure pain threshold (PPT) is the most commonly method to evaluate the degree of nerve sensitization [23]. The PPT decrease indicates peripheral sensitization, and the higher the value, the lighter the degree of sensitization. We selected 4 sites at the diseased knee joint 3 cm from the center of the patella by using a handheld pressure pain meter for PPT measurement. Each site was measured three times to obtain the average value at least 1 min apart.

As to the pain and disability measurement after the injection, all patients were scheduled for follow-up visits at 2, 4 and 8 weeks after treatment. The pain and disability measurement were conducted by different colleague which was blind to the groups. Also, patients were asked to mention adverse reaction.

### Statistical analysis

Statistical analysis was performed using the SPSS for Windows version 25.0 software. Continuous variables were tested by K–S test. The t test was used for measurement data subject to normal distribution among groups, and Fisher exact probability method was used for counting data. The VAS, WOMAC index, and tenderness threshold were compared using repeated measurement analysis of variance. *p* < 0.05 represents a statistically significant difference.

## Results

In this study, 64 patients were enrolled, 1 patient in the IG and 2 patients in the I + PG were excluded for personal reasons and they were lost to follow-up so these patients were not included in the statistical analysis, and 60 patients remained to the end of study. No adverse reactions such as increased pain, bruising and allergy occurred in all patients during treatment and follow-up. Age, sex, BMI and duration of current condition of patients were not significantly different between three groups (Table 1).

**Table 1:** Baseline characteristics of participants

Variables	I + PG (n = 20)	PG (n = 20)	IG (n = 20)	*p *value
Age (years)	61.35 ± 8.32	61.90 ± 5.38	62.75 ± 7.62	0.827
Sex				0.817
Man	9 (45.0)	10 (50.0)	8 (40.0)	
Woman	11 (55.0)	10 (50.0)	12 (60.0)	
BMI (kg/m^2^)	24.83 ± 2.22	23.93 ± 3.47	24.70 ± 1.74	0.493
Duration of symptoms (years)	6 (4.25,6.75)	6 (5.25,7)	6 (5,7)	0.655

### VAS score

**Fig. 3:**
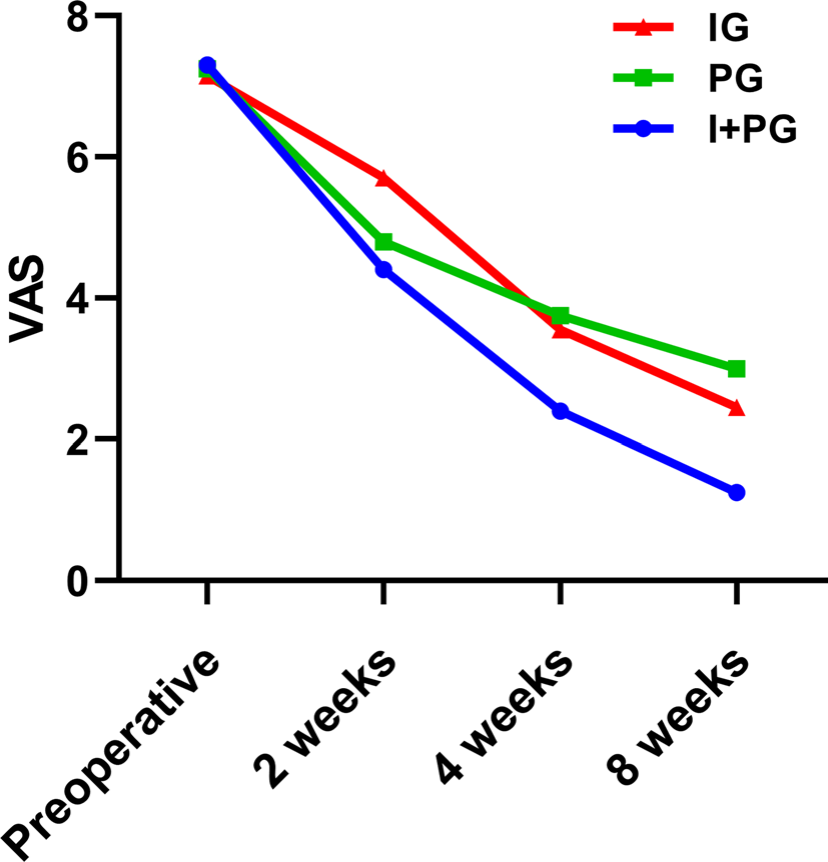
The VAS scores of the three groups at 2, 4, and 8 weeks after treatment

There was no statistically significant with regard to the VAS score before treatment between groups. Compared with the groups, the VAS scores of the three groups of patients decreased at 2, 4, and 8 weeks after treatment compared to before treatment (*p* < 0.05); At the same observation time point among the three groups, after 2 weeks of treatment, the VAS scores of the PG and I + PG were significantly lower than those of IG (*p* < 0.05). However, at 4 and 8 weeks after treatment, the VAS scores of the I + PG were significantly lower than those of the PG and IG, and the difference was statistically significant (*p* < 0.05). There was no significant difference between the PG and IG (*p* > 0.05), (Table 2, Fig. 3).

**Table 2:** Comparison of VAS scores of three groups at different observation time

Groups	Pretreatment	2 weeks	4 weeks	8 weeks	F-Value	*p* Value
I + PG (n = 20)	7.30 ± 0.979	4.40 ± 0.681^a^	2.40 ± 0.598^a^	1.25 ± 0.639^a^	198.566	< 0.001
PG (n = 20) IG (n = 20)	7.25 ± 0.786 7.15 ± 0.933	4.80 ± 1.005^a^ 5.70 ± 0.801^a^	3.75 ± 1.070^a^ 3.55 ± 0.826^a^	3.00 ± 1.257^a^ 2.45 ± 0.686^a^	96.426 141.444	< 0.001 < 0.001
F-Value	0.143	12.572	14.582	19.594		
*p-*Value	0.867	< 0.001	< 0.001	< 0.001		

^a^*p* < 0.05

### WOMAC Score

**Fig. 4:**
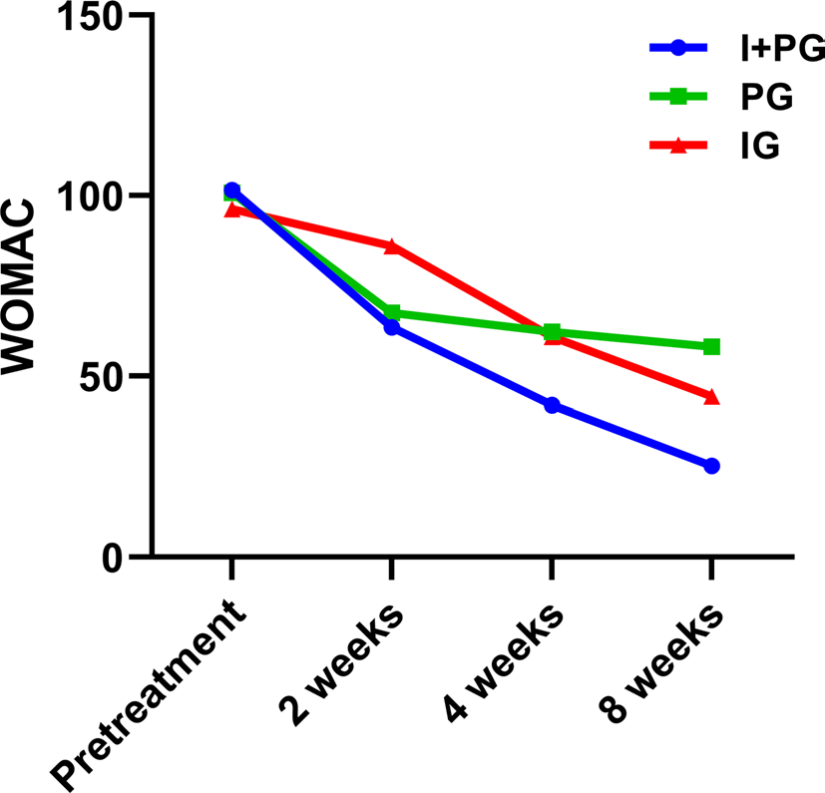
The WOMAC scores of the three groups at 2, 4, and 8 weeks after treatment

There was no statistically significant in regard to the WOMAC score before treatment between groups. Compared within the group, the WOMAC scores of the three groups of patients decreased at 2, 4, and 8 weeks after treatment compared to before treatment (*p* < 0.05); At the same observation time point among the three groups, after 2 weeks of treatment, the WOMAC scores of the PG and I + PG were significantly lower than those of IG (*p* < 0.05). However, at 4 and 8 weeks after treatment, the WOMAC scores of the I + PG were significantly lower than those of the PG and IG (*p* < 0.05). There was no significant difference between the PG and IG (*p* > 0.05), (Table 3, Fig. 4).

**Table 3:** Comparison of WOMAC scores of three groups at different observation time (x ± s)

Groups	Pretreatment	2 weeks	4 weeks	8 weeks	F-Value	*p*-Value
I + PG (n = 20)	101.50 ± 13.9	63.60 ± 15.2^a^	42.05 ± 18.1^a^	25.3 ± 15.7^a^	206.46	< 0.001
PG (n = 20)	100.80 ± 24.6	67.50 ± 22.8^a^	62.25 ± 23.2^a^	58.15 ± 22.8^a^	85.57	< 0.001
IG (n = 20)	96.34 ± 22.5	85.90 ± 21.8^a^	60.95 ± 16.2^a^	44.35 ± 16.5^a^	123.61	< 0.001
F-Value	0.357	6.916	6.759	15.702		
*p-Value*	0.702	< 0.002	< 0.002	< 0.001		

^a^*p* < 0.05

### Pressure pain threshold

**Fig. 5:**
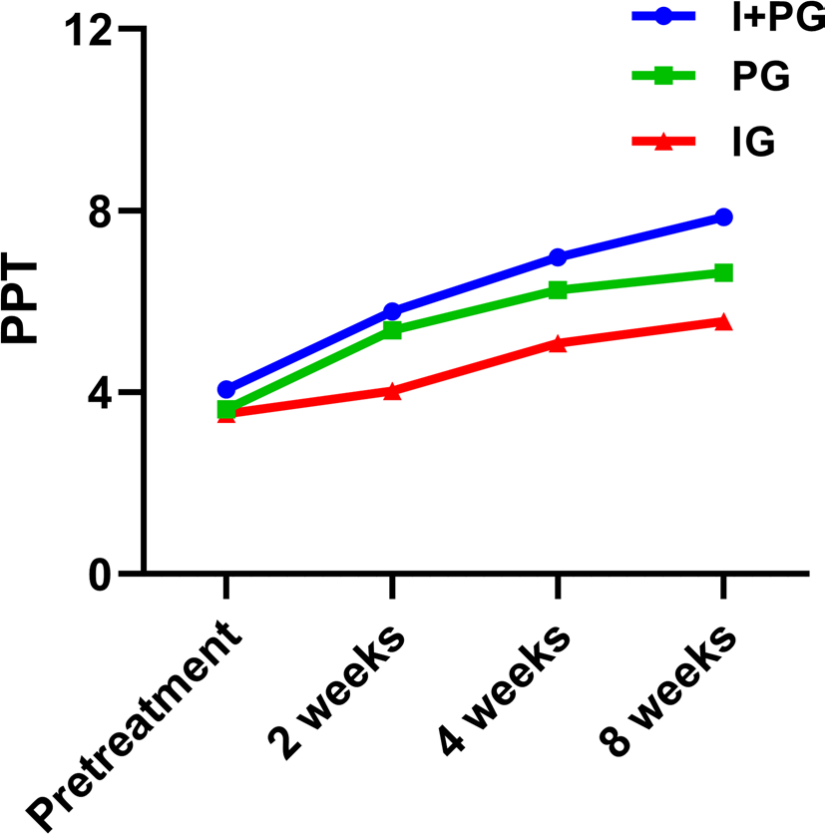
The PPT values of the three groups at 2, 4, and 8 weeks after treatment

Before treatment, there was no statistical difference in the PPT values among the three groups of patients. Compared within the group, the PPT values of the three groups of patients increased at 2, 4, and 8 weeks post treatment compared to pretreatment (*p* < 0.05), At the same observation time between groups, the PPT values of PG and I + PG were significantly improved compared to IG at 2, 4, and 8 weeks after treatment (*p* < 0.05). The PPT values of I + PG were significantly improved compared to PG at 8 weeks after treatment (Table 4, Fig. 5).

**Table 4:** Comparison of PPT values (kg/cm^2^) of three groups at different observation time

Groups	Pretreatment	2 weeks	4 weeks	8 weeks	F-Value	*p*-Value
I + PG (n = 20)	4.060 ± 1.953	5.780 ± 1.674^a^	6.965 ± 1.765^a^	7.855 ± 1.765^a^	80.134	< 0.001
PG (n = 20)	3.620 ± 0.981	5.360 ± 0.841^a^	6.245 ± 0.850^a^	6.630 ± 0.886^a^	55.396	< 0.001
IG (n = 20)	3.520 ± 0.812	4.015 ± 0.816^a^	5.065 ± 0.710^a^	5.560 ± 0.623^a^	32.923	< 0.001
F-Value	0.911	12.223	12.721	18.456		
*p-*Value33	0.408	< 0.001	< 0.001	< 0.001		

^a^*p* < 0.05

## Discussion

In clinic, the most significant symptom of osteoarthritis is pain, which is also the prior goal of the symptomatic treatment [24]. Studies have shown that corticosteroid injections provide short-term pain relief within 4 weeks and is often used during the acute inflammatory phase of KOA patients [25]. However, the long-term efficacy of corticosteroid has not been confirmed, and adverse effects such as ligament rupture also deserve our attention. The main role of hyaluronic acid is to lubricate and protect joints with limitation of anti-inflammatory and analgesic effects [26]. In recent years, researchers have found that the glucose can effectively alleviate pain by injecting inside and outside into the knee joint in KOA. Alketa et al. indicated that the injection of 25% glucose was not only able to improve the pain but also increased the HRQoL score significantly [27]. Reeves et al. showed that the VAS score of KOA patients after intra-articular injection of hypertonic dextrose decreased by an average of 1.69 points compared with pre-treatment, and the sense of misalignment caused by ligament laxity was also significantly improved [28, 29]. Richard et al. found that the use of 20% glucose revealed a decrease in VAS score and Womac index after injection [30]. İsmail et al. applied peripheral nerve injection therapy to KOA patients and found that the average VAS score decreased from 6.7 to 0.76 after injection [16]. Auon et al. selected four sites on the peripheral nerve pathway of the knee joint for subcutaneous injection of glucose. Compared with intra-articular injection, the VAS score and WOMAC index of the peripheral injection group decreased more significantly after treatment. In his opinion, the peripheral injection of glucose is more effective than intra articular injection [31]. In our study, there was no significant difference in the efficacy of those two methods for relieving KOA pain at the 4th and 8th weeks after treatment. In addition, this study found that after 4 and 8 weeks of treatment, the VAS score and Womac index of I + PG significantly decreased compared to PG and IG after the combination therapy. We believed that the combination therapy have better improvement of pain and knee joint function than single PG or IG. At the same time, there was no significant difference in VAS score and Womac index between I + PG and PG at 2 weeks after treatment, but with significantly lower than those in the IG group. This suggested that peripheral nerve injection therapy may have a faster analgesic effect and better early efficacy than intra-articular hyperplasia therapy. This may be related to the different mechanisms between two therapies. Reeves et al. found that injecting hypertonic glucose into the joint could significantly improve the sense of dislocation caused by ligament relaxation [32]. Rabago et al. indicated that intra-articular injection of hypertonic glucose mainly promotes cartilage regeneration, triggers local mild inflammatory reactions around damaged or relaxed ligaments or other joint support structures, triggers healing reactions characterized by the release of growth factors, thereby strengthening ligaments and other joint support structures, improving joint instability, and reducing KOA pain [33]. Liza et al. believed that 5% glucose has the effect of binding to presynaptic calcium channels, inhibiting the release of substance P and CGRP, effectively alleviating neurogenic inflammation and knee pain [34].

Previous studies showed that PPT is one of the common indicators for evaluating nerve sensitization in clinical practice [23]. PPT Measurement of local pain around the affected knee joint is related to peripheral sensitization. A decrease of PPT indicated the peripheral nerve sensitization witch was the higher value with the meaning of lighter symptoms [35]. The results of this experiment showed that the PPT values of the three groups increased after treatment. The PPT of PG significantly increased compared with IG. The PPT degree of elevation varied and the pressure pain threshold of I + PG had the most significantly increase, which indicated that the combined treatment group was the most effective for reducing nerve sensitization. The effect of reducing neural sensitization might related with the peripheral injection therapy of 5% glucose. However, the specific mechanism of reducing sensitization still needs further research in the future.

Ultrasound guidance is able to clearly identify the nerve and its surrounding vascular, muscle, bone and visceral structures. The possible anatomic variation of nerves, blood vessels and surrounding tissues could be identified by scanning before insertion, which helps to design a personalized needle insertion path. The real-time image of needling path has the advantages of adjusting the injection direction and depth to better access the target and reduce the injection number. So, throughout the entire experimental process and follow-up period, the patient had no experienced adverse reactions such as increased pain, bleeding or bruising.

There are still some limitations of this experiment including small sample size and short observation time. The long-term observation of large sample will be conducted to clarify the long-term treatment effect of this therapy. The main purpose of this experiment is the observation of the combined treatment effect. Some objective indicators including imaging indicators could be added to make the experimental results more convincing. In addition, the lack of comparison with other treatments also limits the clinical application of the techniques presented in this paper.

## Conclusion

The ultrasound guided I + PG of 5% glucose seem to be more effective to alleviate pain and improve knee joint function than single therapy in short term. Clinical rehabilitators could clinically try this combination of I + PG to improve clinical symptoms in patients with KOA.

## Data Availability

No datasets were generated or analysed during the current study.
