# Correction: Demonstrating the efectiveness of intra-articular prolotherapy combined with peri-articular perineural injection in knee osteoarthritis: a randomized controlled trial

**DOI:** 10.1186/s13018-024-04845-2

**Published:** 2024-07-09

**Authors:** Yiling Fu, Yukun Du, Jianyi Li, Yongming Xi, Wenbin Ji, Tieshan Li

**Affiliations:** 1 Qilu Hospital of Shandong University (Qingdao), Qingdao, China; 2 The Affiliated Hospital of Qingdao University, Qingdao, China

**Corrrection: Journal of Orthopaedic Surgery and Research (2024) 19:279**

10.1186/s13018-024-04762-4

In this article the affiliation details for Tieshan Li were incorrectly given as ‘Qilu Hospital of Shandong University (Qingdao), Qingdao, China’ but should have been ‘The Afliated Hospital of Qingdao University, Qingdao, China‘.

The original article has been corrected.

